# Responses of Solid Tumor Cells in DMEM to Reactive Oxygen Species Generated by Non-Thermal Plasma and Chemically Induced ROS Systems

**DOI:** 10.1038/srep08587

**Published:** 2015-02-26

**Authors:** Neha Kaushik, Nizam Uddin, Geon Bo Sim, Young June Hong, Ku Youn Baik, Chung Hyeok Kim, Su Jae Lee, Nagendra Kumar Kaushik, Eun Ha Choi

**Affiliations:** 1 Plasma Bioscience Research Center, Kwangwoon University, 447-1, Seoul 139-701, Korea; 2 Institute of Information Technology, Kwangwoon University, 447-1, Seoul 139-701, Korea; 3 Laboratory of Molecular Biochemistry, Department of Life Science, Hanyang University, Seoul 133-791, Korea

## Abstract

In this study, we assessed the role of different reactive oxygen species (ROS) generated by soft jet plasma and chemical-induced ROS systems with regard to cell death in T98G, A549, HEK293 and MRC5 cell lines. For a comparison with plasma, we generated superoxide anion (O_2_^−^), hydroxyl radical (HO·), and hydrogen peroxide (H_2_O_2_) with chemicals inside an *in vitro* cell culture. Our data revealed that plasma decreased the viability and intracellular ATP values of cells and increased the apoptotic population via a caspase activation mechanism. Plasma altered the mitochondrial membrane potential and eventually up-regulated the mRNA expression levels of BAX, BAK1 and H2AX gene but simultaneously down-regulated the levels of Bcl-2 in solid tumor cells. Moreover, a western blot analysis confirmed that plasma also altered phosphorylated ERK1/2/MAPK protein levels. At the same time, using ROS scavengers with plasma, we observed that scavengers of HO· (mannitol) and H_2_O_2_ (catalase and sodium pyruvate) attenuated the activity of plasma on cells to a large extent. In contrast, radicals generated by specific chemical systems enhanced cell death drastically in cancer as well as normal cell lines in a dose-dependent fashion but not specific with regard to the cell type as compared to plasma.

Reactive oxygen species (ROS) are well-known moderators of oxidative damage, playing a role in cell destruction, and activating specific cell death pathways. ROS are free radicals or oxygen containing chemically reactive molecules. ROS can be generated inside a biological system as a natural byproduct of the normal metabolism of oxygen^1^. In normal physiological environments, cells overcome ROS levels by balancing ROS generation with the elimination of ROS by means of a scavenging system. On the other hand, when cell undergo an oxidative stress condition, excessive ROS affects the dynamics of actin cytoskeleton and can damage cellular proteins and DNA, eventually leading to cell death^2^. Tumor cells generally induce high levels of ROS than their normal counterparts. Therefore, cancer cells are more sensitive to the oxidative stress generated by anticancer drug^3^. Over the past few decades, medical personnel have made significant progress in developing many antitumor physical and chemical agents^4^,^5^, such as ionizing radiation^6^,^7^, novel chemical molecules, and other systems that display anticancer activity by means of a ROS-dependent activated pathway of apoptotic cell death, signifying the possible use of ROS as an antitumor approach to treat human cancers. However, many drawbacks remain associated with these therapies due to the resistance and systematic toxicity towards normal cells. The particular ROS types involved in the cell death process remain unclear. Numerous strategies have been employed based on the oxidative stress technique, i.e., the administration of ROS types such as hydrogen peroxide (H_2_O_2_), hydroxyl radicals (HO·), or other ROS-generating chemicals in a tumor bearing animal models. Nevertheless, no successful results were observed, perhaps due to the lack of the selectivity and specificity of the ROS components released in tumor cells, resulting in the induction of side effects^8^.

To overcome these drawbacks, we developed a non-thermal soft air-jet plasma source to induce effective cancer cell apoptosis. Recently, non-thermal plasmas have gained attention in the field of cancer therapeutics. Plasma generally involves a mixture of radicals, reactive species and UV photons. The effects of plasma depend on the reactive species, which are generated in the plasma when biological samples and fluid are brought into contact with the plasma. Many evidences from recent review of literature supported that plasma-induced ROS and RNS effectively kills many types of cancer cells^9^,^10^,^11^,^12^,^13^, and also showed antitumor potential *in vivo*^10^,^13^. Reported work also suggests that non-thermal plasma is capable of producing significant amounts of ozone (O_3_)^14^,^15^, which is known to have very aggressive effects on cells^16^. Oleg et al also claimed role of various plasma and O_3_ in cell death^17^. Ozone generated by plasma have role in the formation of biologically active ROS, RNS and RONS in aqueous media^18^,^19^ which may be responsible for cell death. Subsequently attenuation of the ozone in aqueous media can be possible by the blocking of these species that also effect plasma-exposed cells.

Here, we show that ROS can be delivered to kill cancer cells using two different systems. In this investigation, we conduct our study to check plasma and chemically generated ROS systems effects on cancer and normal cells. We mainly focused on H_2_O_2_, O_2_^−^ and HO· radicals to compare our results with plasma. To induce oxidative stress by a chemical system, xanthine (X) plus xanthine oxidase (XO), which primarily generate O_2_^−^20, was applied in this work. A ROS system for HO· radical formation by the Fenton reaction, which contains CuSO_4_ (C), 1,10-phenanthroline (P) and ascorbic acid (A), was also tested^21^,^22^. However, H_2_O_2_ was directly added to a cell culture from stock solutions at the time of treatment. The present results demonstrated that non-thermal soft-jet plasma leads to cell death with apoptosis characteristics. We also used various ROS scavengers to evaluate specific radical effects by plasma on both cancer and normal cells.

## Results

### The soft-jet plasma and ROS-generating chemical system decreased cell viability

**Figure 1:**
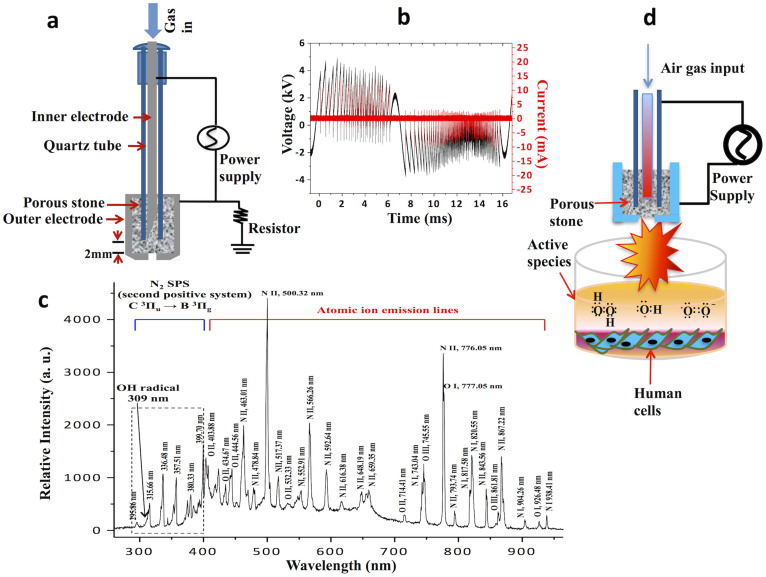
Non-thermal plasma device properties and the experimental set up. (a) Schematic diagram of plasma device (b) Voltage and current characteristics of non-thermal plasma (c) The optical emission spectra (OES) of soft plasma jet (d) Experimental setup of plasma-cell interaction.

**Figure 2:**
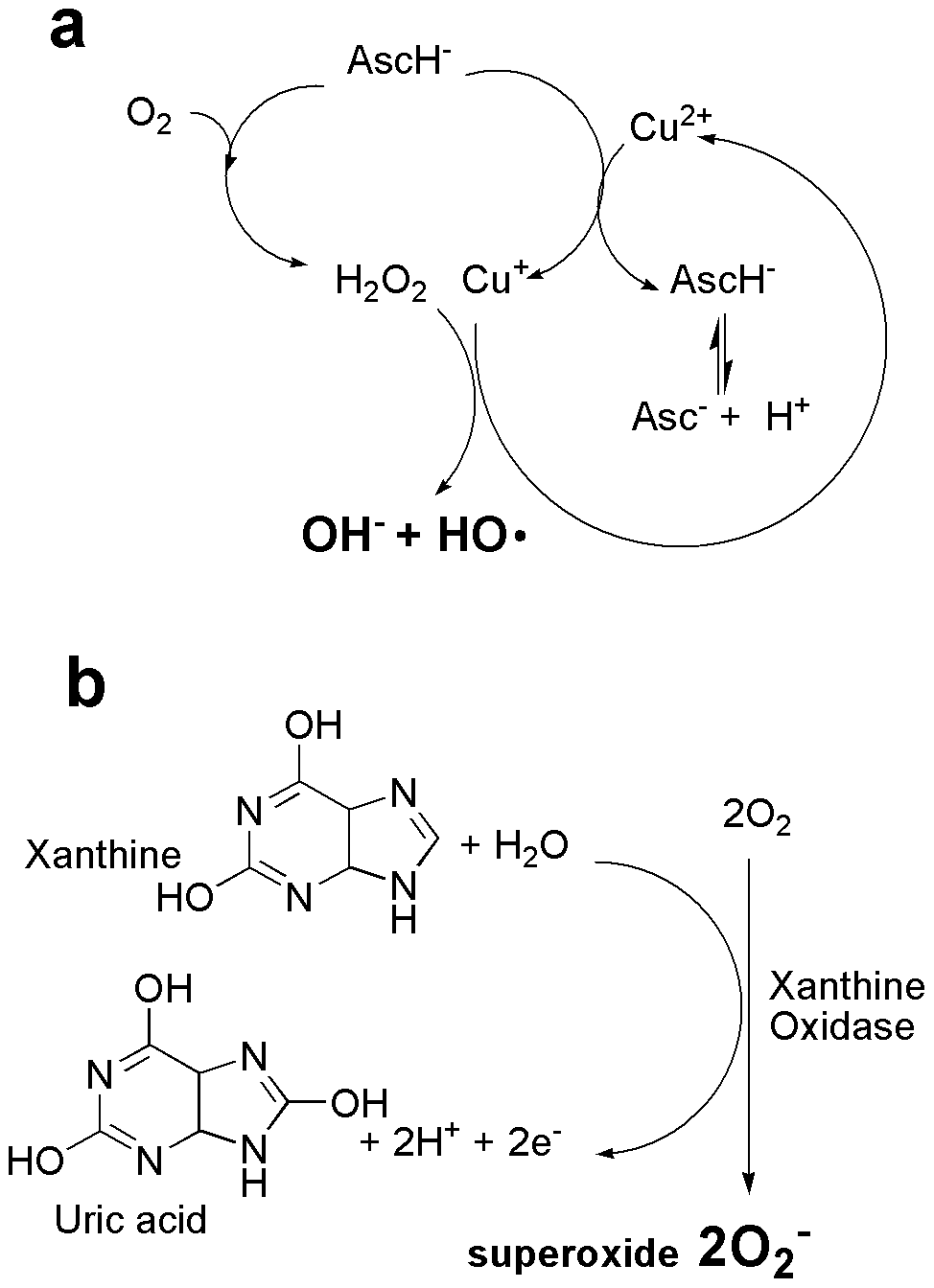
Chemical generated ROS schemes. (a) Formation of hydroxyl radicals (HO·) via Fenton reaction [CuSO_4_, phenanthroline, and ascorbic acid; CPA]. Under aerobic conditions, ascorbate (AscH^−^) not only is involved in the reduction of copper ions (Cu^2+^), but also reacts with O_2_ to produce H_2_O_2_. Hydroxide (OH^−^) and HO· are then yielded in the next Fenton reaction. 1, 10-phenanthroline (P) is used to stimulate HO· formation with Cu^2+^ ions and AscH^−^ (b) Formation of superoxide anion (O_2_^−^) by xanthine (1 mM) plus xanthine oxidase (0.05 U/ml). Xanthine (X) is catalyzed by xanthine oxidase (XO) enzyme and form uric acid and also generates O_2_^−^ in this reaction. This mechanism is based on proposal that the one-electron transfer equilibriums between redox centers of XO enzymes (one molybdenum, one FAD, and two Fe-S centers) are rapid and governed by reduction potentials. During the oxidation of reduced XO electrons transferred to dioxygen. Two O_2_^−^ are produced for each enzyme molecules reoxidized.

**Figure 3:**
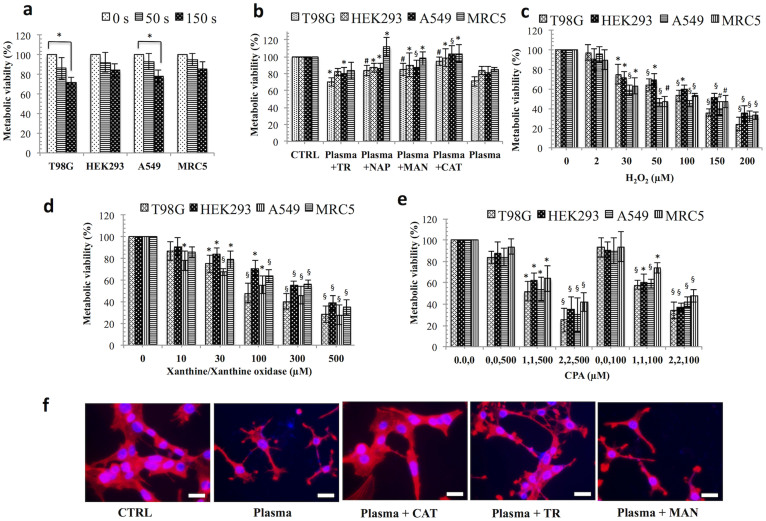
Dose–dependent response of non-thermal plasma and ROS-generating systems on the cancer and normal cells. Viability of cancer and normal cells treated with (a) plasma, (b) plasma plus scavengers, (c) H_2_O_2_, (d) X/XO [xanthine/xanthine oxidase], for O_2_^−^ (e) CPA [CuSO_4_, phenanthroline, and ascorbic acid] for HO· radicals. (f) Immunofluorescence assays using phalloidin rhodamine (5 units/ml, Invitrogen) were performed to visualize the cytoskeleton (F-actin); DAPI was used to label cell nucleus in plasma and plasma plus scavengers treated T98G cells. Each figure has scale bar of 10 μm. Results are expressed as the percentage of living cells compared to control conditions as the mean ± SD (n = 3). Student's *t*-test was performed to controls in plasma, H_2_O_2_, X/XO (O_2_^−^), CPA (HO·), whereas in (a) and (b) plasma and plasma plus scavenger-treated groups were compared to untreated and only plasma-treated group, respectively (* *p* < 0.05, § *p* < 0.01, # *p* < 0.001).

An enhanced level of ROS can trigger irreparable cell death. To investigate which ROS play a main role in initiating cell death, we tested cancer and normal cells in different ROS generating systems. Soft jet plasma (Fig. 1) was used to test combined ROS effect on cell death, whereas for a specific ROS effect, H_2_O_2_, O_2_^−^, and HO· was added to cells separately (Fig. 2). Specific ROS scavengers were also added before plasma exposure to determine the effects of a particular reactive species. First, we examined the viability of T98G, A549, HEK293 and MRC5 cells. Normal cells were severely affected at high dose (such as 5 min exposure) of jet plasma therefore; we selected low dose (50 s) and median dose (150 s) plasma exposure in this study to distinguish our results more clearly between normal and cancer cells. We observed that cancer cells exposed to a 50 s plasma treatment showed a less of an effect than those exposed to a 150 s treatment. However, a 150 s plasma exposure did not induce any significant decrease in the cell viability of HEK293 (*p* = 0.058) and MRC5 (*p* = 0.074) normal cells. A significant inhibitory effect was noted after 150 s plasma exposure of cancer cells, as shown by the inhibition of cell viability up to 28% (*p* = 0.01) and 22% (*p* = 0.02), respectively, in T98G and A549 cells at 24 h, with a range of viability of 72.2% to 78.5% (*p* < 0.05). However, there was no such significant effect after 50 s of plasma exposure on T98G (*p* = 0.16) and A549 (*p* = 0.26) cancer cells when compared to an untreated group (Fig. 3a). We also observed that the cell viability of T98G and A549 cells decrease by 19% (*p* = 0.014) and 22% (*p* = 0.016), respectively, at 72 h (Figure S1, supporting information).

We then tested which particular ROS component is mainly involved in plasma-induced cell death. To rescue cancer and normal cells from the consequential ROS produced in the cell culture after the plasma treatment, we pre-incubated the cells with ROS-specific scavengers. We used sodium pyruvate (NAP) and catalase (CAT) for H_2_O_2_ and tiron (TR) and d-mannitol (MAN) for O_2_^−^, or HO·, respectively. All scavengers were non-toxic on all four-cell lines, whereas TR slightly affected the viability of T98G, HEK293 and A549 cells up to 72 h (Figure S2, supporting information). A plasma treatment with TR slightly increased the viability by 8% and 7% of T98G and A549 cells, respectively, at 24 h when compared to only plasma-treated group, whereas in normal HEK293 and MRC5 cells, viability was increased only to 3% (*p* > 0.05), which was not significant. Using NAP, plasma significantly increased the viability by 12.6% (*p* < 0.001) and 6% (*p* < 0.05) for T98G and A549 cells, respectively, when compared to only plasma-treated group. However, in the presence of CAT, a great increase in the viability was noted, 14.5% (*p* < 0.001) and 22% (*p* < 0.01) for T98G and A549 cancer cells, respectively, when compared to a plasma exposure only. For normal cells, NAP and CAT significantly augmented the viability by 19% (*p* < 0.05). MAN, augmented the viability by 14% (*p* < 0.001), 6.6% (*p* < 0.01), 6.1% *p* < 0.05), and 13% (*p* < 0.05) of T98G, A549, HEK293 and MRC5 cells, all of which were significant (Fig. 3b). We also checked effect of RNS (mainly NO) species on cell death and for this reason we measured cell viability in presence of RNS scavenger 2-(4-Carboxyphenyl)-4,4,5,5-tetramethylimidazoline-1-oxyl-3-oxide (CPTIO) on cells (Figure S3, supporting information). Our data revealed that RNS were also involved in plasma induced cell death.

On the other hand, we also checked the effect of chemical-induced ROS system on cancer and normal cells. To test for different ROS effects on cells, we cultured cells in various ROS-generating systems. H_2_O_2_ was directly added to the cell cultures, and X/XO was used to determine the effect of O_2_^−^, whereas CPA (CuSO_4_, 1,10-phenanthroline, and ascorbic acid) was used to generate HO· radicals via the Fenton reaction, as described in Fig. 2. As expected, the presence of H_2_O_2_ (200 μM), X/XO (500 μM), CPA (2 μM CuSO_4_, 2 μM 1,10-phenanthroline, and 500 μM ascorbic acid) attenuated the viability in a concentration-dependent manner at 24 h in all four-cell lines. For H_2_O_2_, cell viability was sharply decreased by more than 40% significantly, at 50 μM in all cell lines (Fig. 3c). X/XO decreased cell viability by more than 30% (*p* < 0.01) in T98G and MRC5 cells at 100 μM (Fig. 3d). However, CPA attenuated cell viability by 60% (*p* < 0.01) at a concentration of 2, 2,500 μM in all cancer and normal cells. However, when we treated cells with ascorbic acid alone (100 and 500 μM), there was no such significant change in cell viability as compared to untreated control (Fig. 3e). We also observed structural changes (cytoskeleton/nucleus) of plasma and plasma plus scavengers treated T98G (Fig. 3f) and A549 (Figure S4, supporting information) cancer cells.

### Level of intracellular ROS induced by the soft-jet plasma and ROS-generating chemical systems

**Figure 4:**
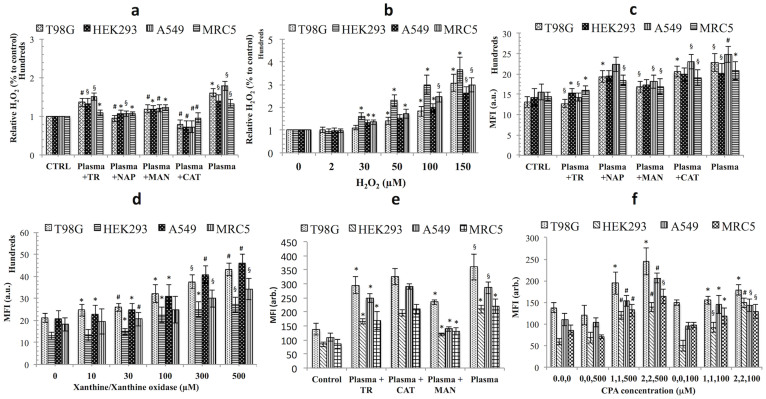
Detection of H_2_O_2_, O_2_^−^ and HO· in plasma and chemical exposed cancer and normal cells. (a, b) H_2_O_2_ level in cells detected with an Amplex Red fluorescent H_2_O_2_ probe in plasma and H_2_O_2_ chemical systems (c, d) O_2_^−^ detection in plasma and chemical systems [xanthine/xanthine oxidase] in cancer and normal cells with a DHE fluorescent probe (1 mM), and (e, f) HO· radical detection using HPF (10 μM) in plasma and CPA [CuSO_4_, phenanthroline, and ascorbic acid] chemical systems by an ELISA plate reader. Results are expressed as the mean ± SD (n = 3). Student's *t*-test was performed to control, whereas in (a), (c) and (e) plasma plus scavenger-treated group was compared to only plasma-treated group (* *p* < 0.05, § *p* < 0.01, # *p* < 0.001).

Because cell cultures (cancer and normal cells) were exposed to different ROS systems from the plasma and the chemicals, we measured the H_2_O_2_, O_2_^−^ and HO· levels inside the cells, using amplex red, dihydroethidium (DHE) and 3-(p-hydroxy-phenyl) fluorescein (HPF) assays. Cell culture responses with respect to each ROS, are shown in Fig. 4 given similar plasma and chemical exposure conditions. In case of only plasma group, the H_2_O_2_ levels were enhanced by 41% to 79% (*p* < 0.05) in all cell lines when compared to an untreated control. Plasma induced a high level of H_2_O_2_ in cancer cells as compared to normal cells, while in the presence of CAT scavengers; as expectedly, the level of H_2_O_2_ decreased from 30% to 60% (*p* < 0.001) in all cell lines as compared to the plasma-treated group whereas NAP decreased H_2_O_2_ levels by 20% to 40% *(p* < 0.05) in all cell lines after being exposed to plasma alone. MAN decreased H_2_O_2_ levels by 10% to 30% (*p* < 0.05) in all cell lines while TR decreased H_2_O_2_ levels only slightly, from 5% to 18%, in all cell lines. Because O_2_^−^ and HO·^23^ both can also form H_2_O_2_ by reaction, therefore their scavengers TR and MAN also H_2_O_2_ decreased levels inside cells. A recent report also claims that scavengers that would be predicted to scavenge particular ROS did not follow the expected behavior of ROS destruction^24^. This may be due to the complex interconversion of ROS due to cellular enzymes and factors—for example, SOD can catalyze the dismutation of to H_2_O_2_^25^. Scavengers showed greatly decreased H_2_O_2_ levels in cancer cells as compared to normal cells (Fig. 4a). On the other hand, in the chemical ROS system (H_2_O_2_), there was a dose-dependent drastic increase in H_2_O_2_ levels with the concentration greater than 30 μM inside all cells (Fig. 4b).

Additionally, we detected intracellular superoxide levels with the plasma and chemical systems (X/XO) when exposed to cancer and normal cells. The plasma group increased a DHE fluorescence intensity (O_2_^−^ level) by 59% to 74% (*p* < 0.01) in cancer cells and 41% to 43% (*p* < 0.05) in normal cells when compared with an untreated group, whereas in the presence of TR, the DHE fluorescence intensity decreased significantly by 42% to 44% (*p* < 0.01) in cancer cells when compared to plasma-treated group, as expected. However, in normal cells, the DHE fluorescence level decreased by only 23% to 24% (*p* < 0.05) by TR. CAT and NAP showed DHE fluorescence decreases by 7% to 15% in cancer cells. It is also reported that DHE somehow also detected some amount of intracellular H_2_O_2_ with O_2_^−^26,27. This may be the possible reason for the decrease in DHE intensity with NAP and CAT scavengers. For MAN, the DHE fluorescence decreased by 26% to 27% (*p* < 0.01) in cancer cells, while in normal cells, the DHE fluorescence intensity was reduced by 14% to 18% (Fig. 4c). When compared to the chemical system (X/XO), the DHE fluorescence intensity increased in a dose-dependent manner. In cancer cells, the X/XO (500 μM) enhanced DHE fluorescence intensity by 104% to 121% (*p* < 0.001), while in normal cells, the intensity increased only by 88% to 108% (*p* < 0.01) (Fig. 4d). Additionally, for a further confirmation of the presence of O_2_^−^ inside the cells, we undertook imaging with DHE, which established that the treatment with plasma increase in intracellular O_2_^−^ levels, without a clear localization within the cell. These results suggest that the O_2_^−^ generation by plasma and X/XO is not localized to a specific part of the cell (Figure S5, supporting information).

Next, we assessed intracellular HO· radicals generation in cancer and normal cells by both ROS-generating systems (plasma and CPA-chemical systems). Plasma significantly enhanced the HPF fluorescence intensity (HO· radicals) by 160% (*p* < 0.01) in both cancer cells, while in normal cells; the HPF fluorescence intensity was increased only by 147% to 153% (*p* < 0.01) when compared to an untreated group. With the addition of a MAN, there were significantly decreased in the HPF fluorescence intensity levels by 34% to 51% (*p* < 0.05) and 40% to 43% (*p* < 0.05) in the both cancer and normal cells, respectively, as compared the plasma-treated group. TR induced an HPF fluorescence intensity decrease only by 12% to 22% in all four-cell lines. In CAT plasma treated group, there was no significant decrease in the HPF fluorescence intensity level in any cell line (*p* > 0.05) (Fig. 4e). On the other hand, the chemical system CPA significantly enhanced the maximum HPF fluorescence intensity at 2, 2,500 μM by more than two-fold (*p* < 0.01) in all cell lines. Ascorbic acid alone did not induce a change in the HPF fluorescence intensity at both concentrations (100 and 500 μM) in all cell lines (Fig. 4f).

### Reduced intracellular adenosine triphosphate (ATP) levels in cells exposed to the plasma and chemical systems

**Figure 5:**
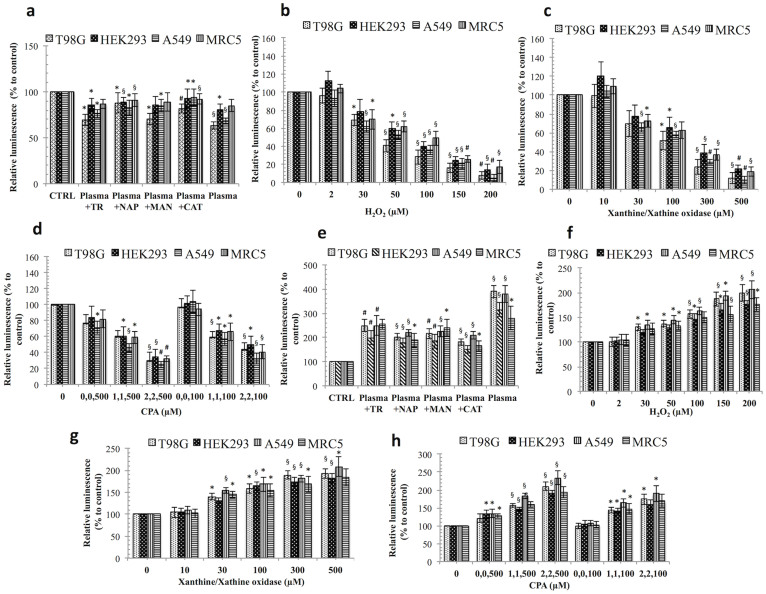
ATP levels and caspase-3/7 activity altered by plasma- and chemical-treated cancer and normal cells. ATP levels in plasma- and chemical-treated cancer and normal cells (a) plasma-treated cells, (b) H_2_O_2_-treated, (c) X/XO [xanthine/xanthine oxidase]-treated, (d) CPA [CuSO_4_, phenanthroline, and ascorbic acid]-treated. Caspase-3/7 activity in cancer and normal cells (e) plasma-treated (f) H_2_O_2_-treated (g) X/XO-treated, and (h) CPA-treated. Results are expressed as the mean ± SD (n = 3). Student's *t*-test was performed to control, whereas in (a), and (e) plasma plus scavenger-treated group was compared to only plasma-treated group (* *p* < 0.05, § *p* < 0.01, # *p* < 0.001).

To determine whether the plasma and chemical-induced ROS systems had an impact on cell energy production, we measured the ATP levels in treated cells in both cases. Plasma attenuated the ATP level by 32% to 35% (*p* < 0.01) in both cancer cells. Significant ATP levels reductions, although less pronounced, were observed even in normal cells by plasma, from 15% to 19%, when compared to an untreated group. In the presence of scavengers (CAT and NAP), ATP levels recovered significantly by 14% to 26% (*p* < 0.05) in cancer cells when compared to the plasma-treated group. For normal cells, the ATP level was recovered only by 6% to 12% by CAT and NAP scavengers. TR increased ATP levels by 5% to 8% (*p* < 0.05) in T98G, A549 and HEK293 cells, while in the MRC5, there was only a 2% (*p* > 0.05) recovery which was not significant, although MAN showed more of a recovery of ATP levels by 7% to 17% (*p* < 0.05) in both cancer cells (Fig. 5a).

In contrast, ATP levels decreased sharply after 30 μM (*p* < 0.05) with the chemically generated (H_2_O_2_ and X/XO) systems in all four-cell lines (Fig. 5b, 5c). However, in CPA, ATP attenuation was significantly pronounced, at 70% to 75% (*p* < 0.01) and 65% to 68% (*p* < 0.01) in cancer and normal cells, respectively, at a maximum concentration of 2,2,500 μM (Fig. 5d).

### Effect of plasma and ROS-generating chemical systems on apoptotic executioner caspases

As shown in Fig. 5e, plasma enhanced the caspase-3/7 activity by 270% to 290% (*p* < 0.01) in both cancer cells, while in normal cells; this level was increased by 180% to 200% (*p* < 0.05) when compared to an untreated control. Increase in caspase activity in normal cell suggests that ROS induced by plasma also have effect on normal cell processes. However, there are some situations when their activation does not lead to death (apoptosis) but its mechanism is still unclear^28^. These caspases are also known to mediate many non-apoptotic functions in cells^29^,^30^. CAT and NAP scavengers reduced the caspase activity by 161% to 209% (*p* < 0.01) in both cancer cells when compared to the plasma-treated group. However, in HEK293 and MRC5 normal cells, CAT and NAP reduced caspases by 137% to 165% and by 91% to 115%, respectively. MAN decreased caspase levels by 154% to 174% (*p* < 0.01) in both cancer cells, while TR had less of an effect on caspases at 130% (*p* < 0.001) for both cancer cells when compared to the plasma-treated group. In addition, MRC5 normal cells showed decreases of 25% and 40% in their caspase activity by CAT and NAP, respectively.

In contrast, the caspase activity level was augmented considerably after 30 μM when using the chemically generated (H_2_O_2_ and X/XO) systems in all four-cell lines (Fig. 5f, 5g). However, in the CPA system, caspase augmentation was significantly marked by 110% to 135% (*p* < 0.01) and 90% to 95% (*p* < 0.01) in the cancer and normal cells, respectively, at a maximum concentration of 2, 2, 500 μM, while ascorbic acid alone also increased caspase activity by 27% to 35% (*p* < 0.01) in A549, HEK293 and MRC5 cells (Fig. 5h).

### Soft-jet plasma effectively induced apoptosis in cancer cells population

**Figure 6:**
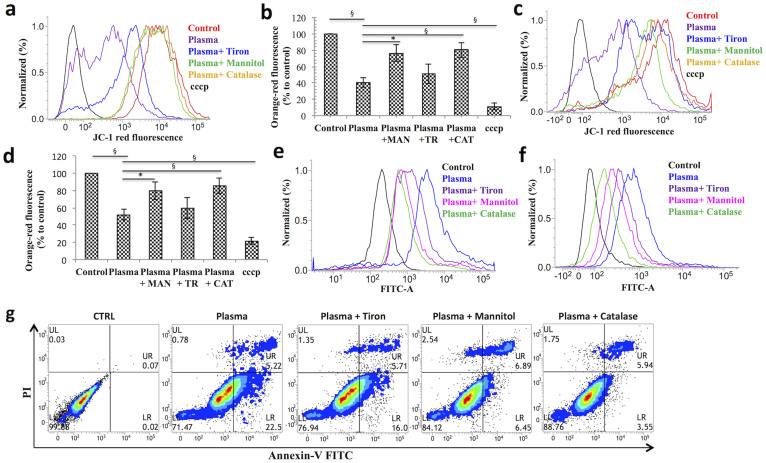
Analysis of apoptotic indicators of plasma and plasma plus scavengers treated cancer cells. (a) Flow cytometric band shift plot of mitochondrial membrane potential in T98G cells, where cccp was used as a positive control. (b) A bar graph of calculated JC-1 red fluorescence percent intensity changes of T98G cells (c) Flow cytometric band shift plot of mitochondrial membrane potential in A549 cells. (d) A bar graph of JC-1 red fluorescence calculated percent intensity changes of A549 cells. Apoptotic cell death was assessed using annexin V-FITC/PI staining and flow cytometry. Stained cells can be discriminated cells into four groups, i.e., the viable (annexin V− PI−), early apoptosis (annexin V+ PI−), late apoptosis (annexinV+ PI+) and necrotic (annexin V− PI+) groups. (e) A plot of T98G cells apoptotic population treated by plasma and plasma plus scavengers. (f) A plot of A549 cells apoptotic population treated by plasma and plasma plus scavengers. (g) Contour plot of T98G cells population treated with plasma and plasma plus scavengers. Results are expressed as the mean ± SD (n = 3). Student's *t*-test was performed to control, whereas in plasma plus scavenger-treated group was compared to only plasma-treated group (* *p* < 0.05, § *p* < 0.01, # *p* < 0.001).

BAX translocation to the mitochondria results in the opening of mitochondrial permeability transition pores and attenuates the mitochondrial membrane potential (MMP)^31^. Therefore, we subjected plasma and plasma plus scavengers treated T98G and A549 cells to the JC-1 cationic dye in a FACS analysis to examine whether plasma results in a drop in the MMP. For positive control, we used a proton ionophore carbonyl cyanide 3-chlorophenylhydrazone (cccp). Fig. 6a, 6c show the band shift phenomenon for the red fluorescence of JC-1 observed after 24 h of incubation in the T98G and A549 cells, respectively. To calculate the relative change of the MMP, we assessed the differences in the red fluorescence intensity between a negative control and a positive control; the calculated percent intensity changes are plotted in Fig. 6b, 6d for T98G and A549 cells, respectively. Only plasma group demonstrated a 48% to 59% (*p* < 0.01) significant decrease in the JC-1 orange-red fluorescence in both cancer cells as compared to an untreated control. In contrast, the presence of CAT and MAN scavengers increased the red fluorescence of JC-1 by 27% to 45% in cancer cells when compared to the plasma-treated group. However, in the TR case, there was a slight change in the JC-1 fluorescence level by 7% to 10% in both cancer cells. These results displayed increased JC-1 red fluorescence levels with a low MMP rate, confirming that plasma-induced apoptosis also via the mitochondrial death pathway.

To check whether cells undergo apoptosis, plasma-and plasma plus scavengers treated cancer cells were stained with annexin V-FITC and PI and analyzed by FACS system. Exposure to plasma only for 150 s resulted in an increase in the apoptotic population in T98G cells by 27.8% (*p* < 0.05) as compared to an untreated control, whereas CAT and MAN led to a decrease in the apoptotic population by 14% to 17% (*p* < 0.05) in T98G cells when compared to the plasma-treated group. TR was also found to decrease the apoptotic population in T98G cells by 6%, which is not significant (*p* > 0.05) as compared to the plasma-treated group, as shown in Fig. 6e, 6g. For the A549 cells, similar profile was observed (Fig. 6f, Figure S6, supporting information).

### A plasma treatment triggers a mitochondrial apoptotic intrinsic pathway involving the regulation of Bcl-2 family members

Cancer cells escape apoptosis by overexpressing anti-apoptotic proteins or through the compact expression of pro-apoptotic proteins. Bcl-2 family proteins, including anti-apoptotic members (such as Bcl-2) and pro-apoptotic members (such as BAX and BAK), have been confirmed as among the crucial factors, playing a key role in apoptotic cell death^32^. It is well known that the translocation of BAX from cytosol to mitochondria is required for BAX and BAK oligomerization, and the release of mitochondrial proteins^33^.

**Figure 7:**
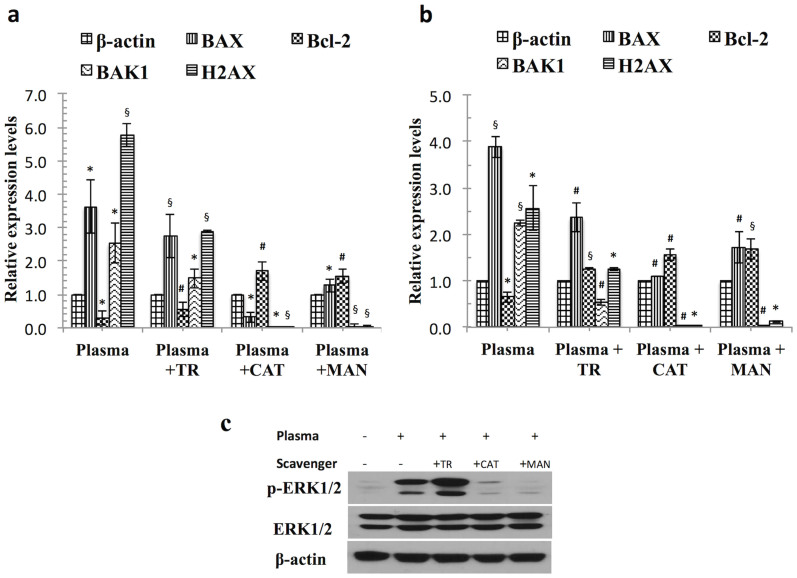
Soft-jet plasma treatment triggers a mitochondrial apoptotic intrinsic pathway and activated ERK/MAPK protein levels. (a) RT-PCR quantitation of mRNA levels for Bcl-2 family members in T98G cells (b) RT-PCR quantitation of mRNA levels Bcl-2 family members in A549 cells. Results are expressed as the mean ± SD (n = 3). Student's *t*-test was performed to control, whereas plasma plus scavenger-treated group was compared to only plasma-treated group (* *p* < 0.05, § *p* < 0.01, # *p* < 0.001). (c) Western blot analysis was performed using antibodies against p-ERK in T98G cancer cells. Cropped blots were used here and uncropped images of blots are shown in supplementary Figure S7, gels/blots have been processed under the same experiment conditions.

In a further study of the possible mechanism underlying plasma-induced cancer cell apoptosis at 24 h, we determined the mRNA gene expressions of Bcl-2, BAX, BAK1 and H2AX by real time quantitative PCR analysis. Our results showed that the expression of these genes increased by 3.6 to 3.9 fold and 2.2 to 2.5 fold with regard to BAX and BAK1, respectively, whereas the Bcl-2 level decreased by 0.4 to 0.8 fold i.e., significant (*p* < 0.05), as compared to β-actin in both cancer cells. As expected, H2AX genes were also significantly highly expressed after the plasma treatment, by 2.6 to 5.7 fold (*p* < 0.05) in both cancer cells (Fig. 7a, 7b). H2AX becomes phosphorylated upon serine 139, known as gamma-H2AX; resulting in DNA Double-strand breaks (DSB). In the presence of CAT and MAN, significantly decreased expression of BAX, BAK1 and H2AX genes by 2.7 fold (*p* < 0.001), 2.2 fold (*p* < 0.001) and 2.5 fold (*p* < 0.05), respectively, were noted in A549 cells, whereas in T98G cells, CAT and MAN decreased the expression of BAX, BAK1 and H2AX genes by 2.4 fold (*p* < 0.05), 2.5 fold (*p* < 0.05) and 5.7 fold (*p* < 0.01), respectively, when compared to the plasma-treated group. In addition, Bcl-2 was significantly increased by 1 to 1.5 fold (*p* < 0.001) in both cancer cells, as compared to the plasma-treated group. We also observed less of a decrease in the levels of the pro-apoptotic genes (BAX, BAK1 and H2AX) by TR scavengers as compared to groups treated with CAT and MAN in combination with plasma.

### ERK1/2-MAPK is involved in cancer cell death in response to plasma exposure

MAPKs are involved in the regulation of apoptotic cell death in response to various stimuli. The temporary activation of the ERK cascade leads to cell survival and proliferation, although lasting ERK activation results in an apoptotic response in malignant gliomas as reported previously^34^. To confirm the potential involvement of MAPK in plasma-induced cell death, we primarily measured changes in ERK1/2 activity after a plasma treatment in T98G cancer cells at 24 h incubation. As shown in Fig. 7c, plasma-treated cells showed a dramatic increase in the phosphorylation of ERK1/2 activity when compared to an untreated group, but a gradual down-regulation of phosphorylated ERK1/2 levels in the presence of scavengers. However, plasma with TR also increased phosphorylated ERK1/2 levels. This may have stemmed from the fact that TR was not able to neutralize the effect of plasma significantly while also inducing toxicity in cells. The total ERK1/2 cellular levels remained constant. Our results indicate that ERK1/2 was involved in plasma-induced cell death in T98G cancer cells.

## Discussion

Two kinds of ROS generating systems were utilized in this study to find out some clue about particular ROS species ultimately contributing to cancer cell death *in vitro*. Various types of non-thermal plasma offers the ability to deliver ROS directly or indirectly into living tissues, implying its feasibility as an innovative device for use in cancer therapy by endoscopic or branched organ targeting treatment technology^35^,^36^,^37^,^38^,^39^. Plasma generated ROS activate the cancer cell apoptosis by decreasing metabolic viability, intracellular ATP levels. Apoptosis induction is an effective way to stop the progress of tumor growth *in vitro*. Apoptotic cells show signs of typical apoptotic feature including various morphology changes such as chromatin condensation, cell blabbing^40^. Mitochondria are primarily affected early in the apoptotic process and are now thought to act as central coordinators of cell death^41^. MMP loss (Δψm) has a key role in activating mitochondrial intrinsic apoptosis pathway and these changes in mitochondria are closely regulated by Bcl-2 family genes expression^42^. It was noted here that the plasma generated reactive species such as HO·, O_2_^−^, H_2_O_2_ can activate mitochondrial-mediated apoptosis by the change in MMP and simultaneously up-regulates pro-apoptotic genes and down-regulates anti-apoptotic genes for activation of caspases^43^ and eventually cell undergoes apoptosis. In addition, H2AX mRNA gene expression levels were also increased after plasma treatment in cancer cells. H2AX is a DNA damage marker that can be activated in response to DNA damage. Activated histone can point toward to upstream molecules, such as ataxia telangiectasia-mutated and other-related proteins^44^. Increased expression levels of p-ERK also regulated the anti-cancer effect of plasma. Recently, some evidences support the potential involvement of ERK1/2/MAPK activation in cancer cell death induced by anticancer drug^45^. To identify the role of specific ROS in cell death, we used specific ROS scavengers before the plasma treatment. Based on our data, it can be expected that cell death was more likely to be associated with H_2_O_2_ and HO· components and was least affected by O_2_^−^. There were significantly fewer number of cells rescued from the effect of plasma in the TR case. This may be due to the reasonable level of the toxicity of the TR on these cells. We observed a significant protection effect of cells by CAT and MAN scavengers against a plasma treatment, indicating strong relationship between the generation of HO· radicals and subsequent formation of H_2_O_2_, by soft-jet plasma. The formation of some fraction of H_2_O_2_ from HO· radical species induced by plasma in cell cultures or bio-solutions is possible. However, the fraction of cells that were affected by HO· radical species along with H_2_O_2_ or HO· and H_2_O_2_ alone remain unclear. However, further study is required to study these reactive species effects on cell death more in detail. There are many possibilities by which these reactive species have direct or indirect effect on cells inside aqueous medium. Few can have direct effect or can react to form more stable long-lived reactive species that can also have activity against biological samples. It is mentioned previously that H_2_O_2_, O_2_^−^ and HO· have roles in the formation of RONS, which effect biological activity^18^,^19^,^23^,^46^,^47^. So there are also possibilities by which formation of RONS is affected if we block specific reactive oxygen or nitrogen species. Therefore, blocking these reactive species not only affects their biological activity, but also alters activity of other species such as RONS.

Taken together, we concluded here that particular types of ROS scavenging by antioxidants might provide a key to our understanding of activated ROS-triggered signaling pathways in cancer cell death. Our data demonstrated an interaction between plasma and scavengers in ambient cell cultures. Multiple antioxidant defense systems, which become active in cells, interfere with responses expected from the mechanism of action of scavengers in cell systems. Plasma-induced cancer cell death arose due to the activation of the mitochondrial intrinsic pathway. Plasma also activates the ERK1/2/MAPK oxidative stress pathway, which also plays an important role in apoptotic cell death whereas scavengers (CAT and MAN) showed attenuation of these cellular activities as induced by plasma. On the other hand, when compared chemically generated ROS systems, these systems (for H_2_O_2_, O_2_^−^, and HO·) drastically increased cell death in a dose-dependent fashion. These systems were not specific with regard to the cell type, i.e., cancer and normal cells. There was a possibility of direct toxic effect of the chemicals used to generate these systems. Whereas plasma induced oxidative stress depend upon the size of changes, moderate or more severe oxidative stress could trigger apoptosis. A cumulative effect of different ROS induced by the plasma also affects normal cells, which have less inhibitory effect than on cancer cells.

**Figure 8:**
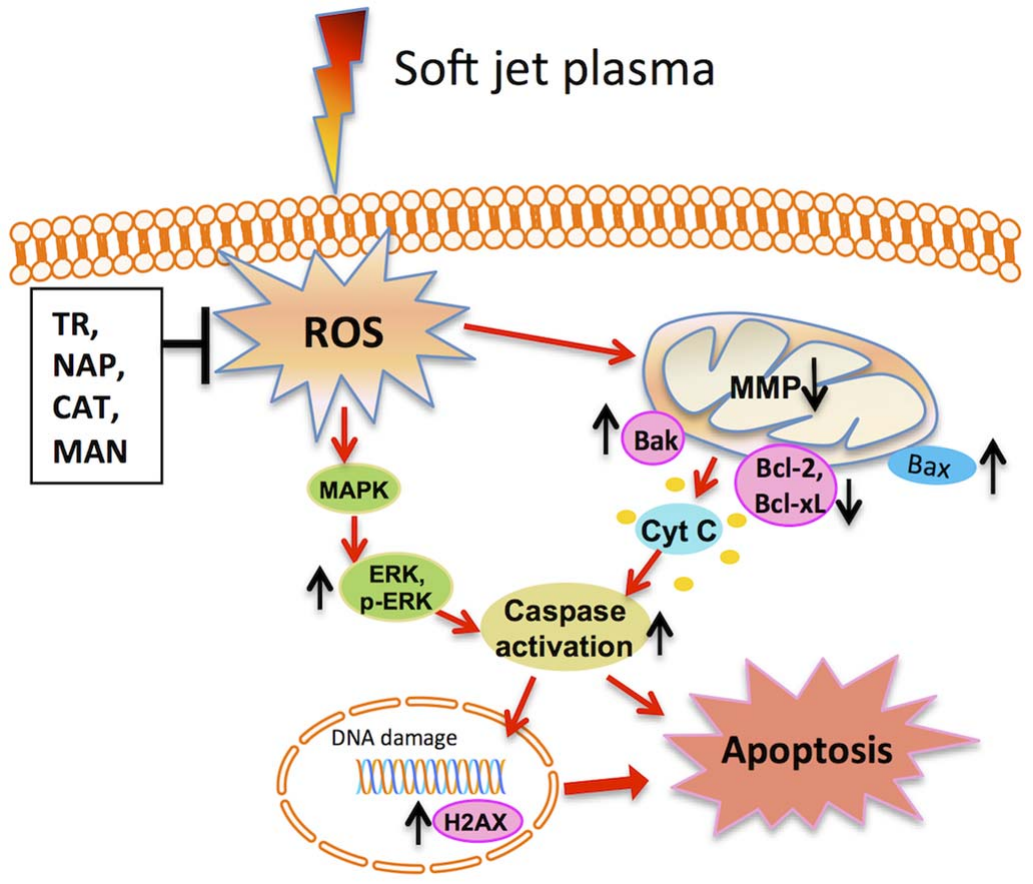
The molecular mechanism of soft-jet plasma induced cancer cell apoptosis via mitochondrial intrinsic pathway and ERK/MAPK activation.

In summary, we showed the ability of soft jet plasma to enhance cancer cell death *in vitro* by mitochondria-mediated apoptosis and this sensitization could be achieved by ROS- (H_2_O_2_ and HO· radical mainly) mediated DNA damage and activation of ERK1/2/MAPK pathway (Fig. 8). This report may provide some basic information with which to understand the essential mechanisms relevant to ROS in cancer cell death.

## Methods

### Cell culture and reagents

T98G (glioblastoma), HEK293 (normal embryonic kidney), A549 (lung adenocarcinoma) and MRC5 (normal lung fibroblast) cell lines were purchased from the Korean cell line bank (Seoul, Korea). All cells were grown in DMEM high glucose (Hyclone, USA) media supplemented with 10% fetal bovine serum and 100 U/ml penicillin–streptomycin (Hyclone, USA) at 37°C with 5% CO_2_. 4, 5-Dihydroxy-1, 3-benzenedisulfonic acid disodium salt monohydrate (tiron), d-mannitol, sodium pyruvate, catalase, coppersulphate, 1, 10-phenanthroline, ascorbic acid and polyclonal anti-β-actin antibody werepurchased from Sigma-Aldrich, Korea. Polyclonal antibodies tophospho-ERK1/2 and ERK1/2 were purchased from Cell Signaling Technology, Inc. USA.

### Experimental device configuration, optimized conditions, and plasma treatment

Fig. 1a demonstrates the experimental configuration of the soft plasma-jet system at atmospheric pressure, consisting mainly of a high-voltage power supply, electrodes, and dielectrics. The porous stone used here has a porosity of 30% and a pore diameter of 150 μm; it served, as a dielectric vehicle among stainless steel electrodes to induce micro-discharges and decrease the gas temperature. The output voltage (1.8 kV) and current (15 mA) waveforms have a profile with an average power of 3 W (Fig. 1b). The air gas flow rate was 1 L/min, and the system was operated in open air with across a 1 mm gas hole. During the air plasma operation, the length of the plume was approximately 3–4 mm at a gas flow rate of 1 L/min. Optical emission spectroscopic measurements were taken with a charge-coupled device spectrometer (HR4000, Ocean optics) and optical fiber (QP400-2-SR) with a diameter of 600 μm (Fig. 1c). The distance between the tip of the plasma discharge device and the media surface was fixed at 3 mm and the depth of DMEM is about 4 mm from the upper surface of media (Fig. 1d).

### ROS generating chemical system

To generate ROS *in vitro*, standard procedures were performed as described in previous reports^20^,^21^,^22^. Chemically generated ROS produced by the experimental techniques were as follows: H_2_O_2_ (30% stock), superoxide anion (O_2_^−^) (1 mM xanthine and 0.05 U/ml of xanthine oxidase), and a hydroxyl radical- HO· (CuSO4, 1,10-phenanthroline, and ascorbic acid; CPA, Fenton reaction). Concentrations were used in the following ranges: H_2_O_2_ (2–200 μM), 10–500 μM (X/XO) and CuSO_4_ (0–2 μM), 1,10-phenanthroline (0–2 μM) and ascorbic acid (0–500 μM) (Fig. 2).

### ROS scavenging

To investigate the particular ROS effect on cells, we used scavengers in the plasma- induced ROS system. The scavengers utilized here were 4, 5-Dihydroxy-1,3-benzenedisulfonic acid disodium salt monohydrate (tiron,10 μM), d-mannitol (25 mM), sodium pyruvate (10 mM), and catalase (60 U/ml). The toxicity levels of all scavengers were tested beforehand to avoid cell toxicity (Figure S2, supporting information). ROS scavengers were added to the wells 2 h before the plasma exposure.

### Cells viability test

MTT assays, a generally accepted colorimetric assay, which can be used to measure the mitochondrial activity, were used here to check cell viability. Scavengers were added to each well of cell culture plate as described above and were treated with plasma (50 s and 150 s) followed by incubation for 24, 48 and 72 h at 37°C. Chemically (H_2_O_2_, O_2_^−^, HO·) exposed cells were also incubated for 24 h without the presence of scavengers. After the desired period of time, MTT assay was performed and the samples absorbance were monitored using plate reader (Synergy HT, Biotek) at 540 nm and the data was analyzed as described previously^48^,^49^,^50^,^51^.

### Intracellular hydrogen peroxide (H_2_O_2_) assay

To determine the existence of H_2_O_2_ after scavenging in the plasma system and in the chemically generated H_2_O_2_ system, H_2_O_2_ assays (Amplex Red; Invitrogen) were performed. Cells were exposed to the plasma (150 s) and chemical treatment under similar conditions. The dye (50 μM of Amplex® Red reagent and 0.1 U/ml of Horseradish peroxidase (HRP) in Krebs–Ringer phosphate; Invitrogen) was added to each well of the plasma-treated and chemically generated ROS systems to a final volume of 100 μl and samples were incubated for 30 min in the dark. Fluorescence was monitored at ex/em values equal to 485 nm/580 nm.

### Intracellular superoxide anion (O_2_^−^) assay

To investigate the superoxide anion inside the cells, the fluorescent probe Dihydroethidium (1 mM DHE; Invitrogen) was used. DHE reacts mainly with O_2_^−^ to form an oxidized fluorescent product. After getting into the cell, this probe is oxidized to ethidium^52^. DHE was added to cells after the plasma and chemical treatment. The plate was incubated for 30 min in a dark condition. Fluorescence readings were detected using a plate reader with ex/em at 485 nm/528 nm. For a visual observation of the intracellular superoxide, fluorescence-stained cells (2 × 10^4^) were then visualized under a fluorescent microscope (Ti-U, Nikon).

### Intracellular hydroxyl radical (HO·) assay

To determine the quantity of hydroxyl radical generation inside the cells, the fluorescent dye 3-(p-hydroxy- phenyl) fluorescein (HPF, Invitrogen) was used at a concentration of 10 μM. Briefly, HPF was pre-incubated in cell media in a dark condition, protected from light for 30 min, and then exposed to the plasma and chemical treatment. Fluorescence readings were detected using a plate reader with ex/em at 485 nm/528 nm.

### Cytoskeleton arrangement analysis

Actin fibers and the nucleus were stained after 24 h of plasma treatment. Cells grown on cover glass were washed with DPBS (Welgene, Korea), fixed with 4% paraformaldehyde (Gentic, USA) for 20 min, and permeabilized by 0.1% of Triton X-100 (Sigma-Aldrich, Korea) in PBS for 25 min at room temperature. After washing with DPBS, actin fibers were stained by phalloidin dye (5 units/ml, Phallotoxins; Invitrogen) for 30 min at room temperature. After washing thrice with DPBS, the samples were mounted with the Prolong gold anti-fade reagent with DAPI (Molecular probes; Invitrogen) and observed under ×60 magnification with a fluorescence microscope (Ti-U, Nikon).

### Cellular ATP level detection

The intracellular ATP levels of the plasma- and chemical-treated cells (2 × 10^5^) were measured using EnzyLight™ ATP assays (Bioassays Systems, CA) according to the manufacturer's instructions. The luminescence levels were measured using a luminometer (Synergy HT, Biotek) and quantities relative to ATP standards.

### Apoptotic executioner assay (caspase-3/7 activity)

Caspase-3/7 activities were measured in triplicates using the Caspase-Glo® 3/7assay kit (Promega) according to the manufacture's protocol. The luminescences of the caspase activity in cells were measured using a luminometer.

### Measurement of the mitochondrial membrane potential (MMP or Δψm)

JC-1 cationic dye (MitoProbe™ JC-1 Assay Kit - Life Technologies) was used to analyze Δψm by means of a flow cytometric analysis assay, as previously described^53^. Briefly, plasma-treated cells were harvested and stained with 2 μM of JC-1 for 30 min at 37°C in the dark, after washed with DPBS twice, immediately analyzed by FACSVerse system (BD Biosciences, NJ, USA). Data were analyzed using the FACS suite software. Results were expressed as the fraction of the cells with decreased red fluorescence, indicating the loss of Δψm.

### Quantification of apoptosis

To determine cell death, annexin V-FITC and propidium iodide staining was performed using a BD Pharmingen™ FITC Annexin V Apoptosis Detection Kit I according to the manufacturer's protocol (BD Biosciences, USA). In brief, cells were treated with plasma and then further incubated for 24 h, after which they were harvested, washed with DPBS, and stained with Annexin V-FITC and propidium iodide (PI). Early and late apoptosis were quantified according to the manufacturer's instructions using FACSVerse system and FACS suite software.

### Real-time reverse transcriptase-polymerase chain reaction (real-time RT-PCR)

Total RNA was extracted from cells using the Trizol reagent (Invitrogen) and cDNA was synthesized using (Superscript II reverse transcriptase kit, Invitrogen). Significant changes in the mRNA expression of the genes of interest were calculated by means of real-time RT-PCR. Real-time PCR was performed on a CFX96™ Real-Time System with a, BioRad machine with the IQ SYBR Green Supermix (BD Biosciences). Primers were designed using Primer3 program (v.0.4.0)^54^. The primer sequences are listed in Table S1, Supplementary information. The PCR conditions consisted of a 3 min hot start at 95°C, followed by 40 cycles of 10–15 s at 95°C and 30–60 s at the optimized temperature. The levels of gene expression relative to β-actin were determined as previously described^55^.

### Western blot analysis

A western blot analysis was performed as described in a previous report^56^. The proteins were separated by SDS-PAGE and transferred to a nitrocellulose membrane. The membrane was blocked with 5% skim milk in TBST for 1 h at room temperature and incubated with the respective primary antibodies overnight at 4°C. After repeated washings with TBST, the membranes were incubated with the HRP-conjugated secondary antibody for 2 h at room temperature and the proteins were visualized by enhanced chemiluminescence procedures (Amersham) using the manufacturer's protocol. Equal loading was confirmed using an antibody against β-Actin.

### Statistical Analysis

All of the resulting values were expressed as the mean ± standard deviation (S.D.) of three independent tests. Statistical analysis was performed using the Student's *t*-test to check the significance levels (*p < 0.05, §*p* < 0.01, # *p* < 0.001). In statistics, the *p*-value (helps to determine significance level) is the probability of obtaining the observed sample results, when the null hypothesis is actually true.

## Author Contributions

N.K., N.K.K., C.H.K., S.J.L. and E.H.C. designed/supervised the study. N.K., N.U. and N.K.K. performed the study, analyzed the data and wrote the manuscript. N.K.K., K.Y.B., S.J.L. and E.H.C. contributed the materials. Y.J.H. and G.B.S. provided assistance with plasma design and provide the OES spectra, voltage-current characteristics.

## Supplementary Material

Supplementary Information Supporting information
