# Supplementary material for: Responses of Solid Tumor Cells in DMEM to Reactive Oxygen Species Generated by Non-Thermal Plasma and Chemically Induced ROS Systems
Source: Sci Rep. 2015 Feb 26;5:8587. doi: 10.1038/srep08587 (PMC4341198; doi:10.1038/srep08587)
Supplement: Supplementary Information — Supporting information [file srep08587-s1.pdf]

Supporting Information

Responses of Solid Tumor Cells in DMEM to Reactive Oxygen Species Generated by Non-Thermal Plasma and Chemically Induced ROS Systems

Neha Kaushik,[†1] Nizam Uddin,[3] Geon Bo Sim,[1] Young June Hong,[1] Ku Youn Baik,[1] Chung Hyeok Kim,[2] Su Jae Lee,[3] Nagendra Kumar Kaushik,[1†]* Eun Ha Choi[1]*

[1]*Plasma Bioscience Research Center, Kwangwoon University, Seoul 139-701, Korea*

[2]*Institute of Information Technology, Kwangwoon University, Seoul 139-701, Korea*

[3]*Laboratory of Molecular Biochemistry, Department of Life Science, Hanyang University, Seoul 133-791, Korea*.

Corresponding author: kaushik.nagendra@kw.ac.kr and ehchoi@kw.ac.kr

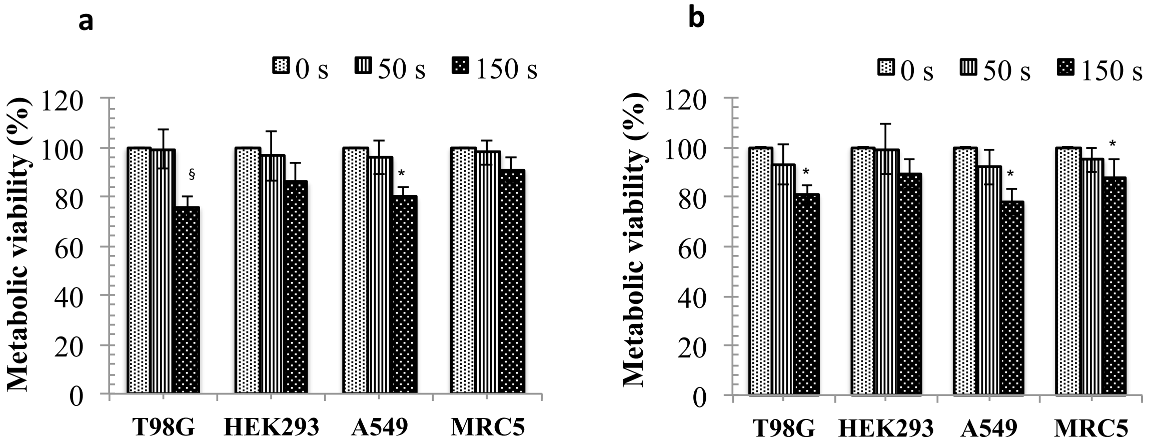

Figure S1. Dose–dependent response of non-thermal plasma. Viability of cancer and normal cells treated with plasma at (a) 48 hour (b) 72 hour. Results are expressed as the percentage of living cells compared to control conditions as the mean ± SD (n=3). Student's *t*-test was performed to controls (* $p<0.05$, § $p<0.01$, # $p<0.001$).

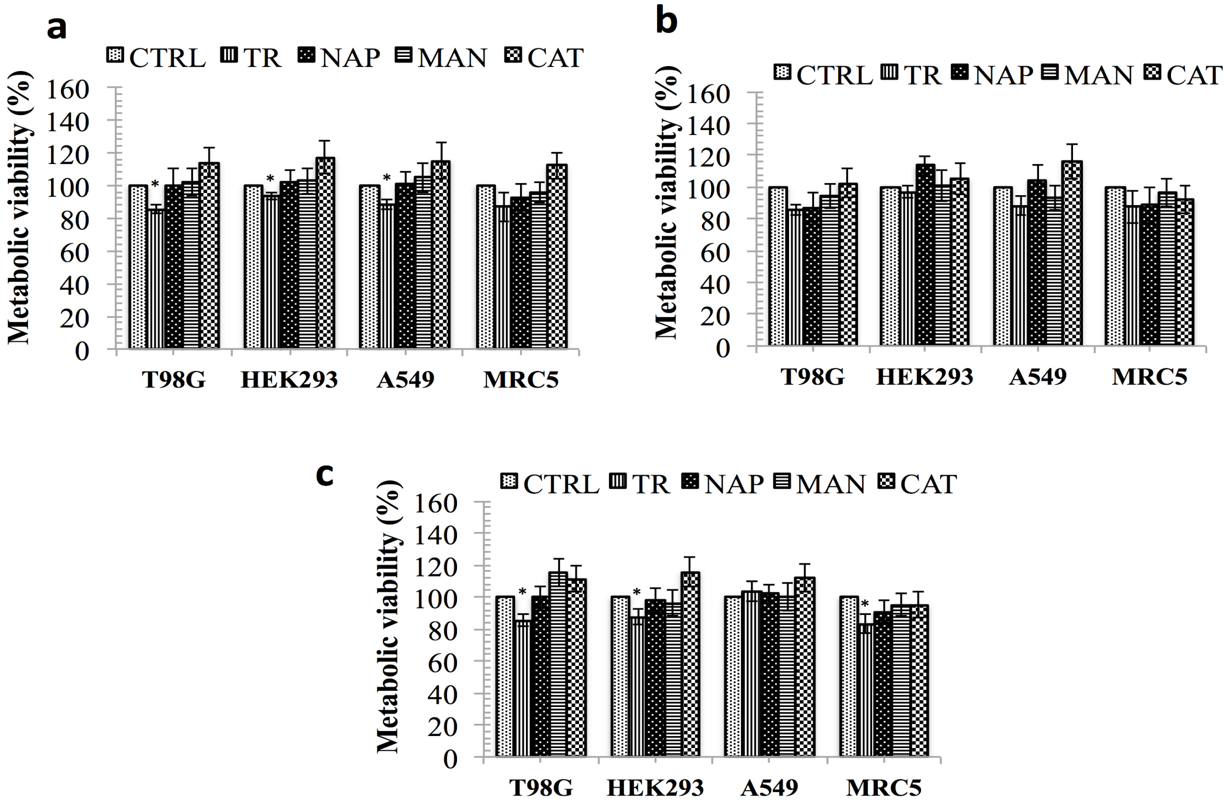

Figure S2. Cytotoxicity of scavengers on cancer and normal cells at (a) 24 hour (b) 48 hour (c) 72 hour. Results are expressed as the percentage of living cells compared to control conditions as the mean ± SD (n=3). Student's *t*-test was performed to controls (* p<0.05, § p<0.01, # p<0.001).

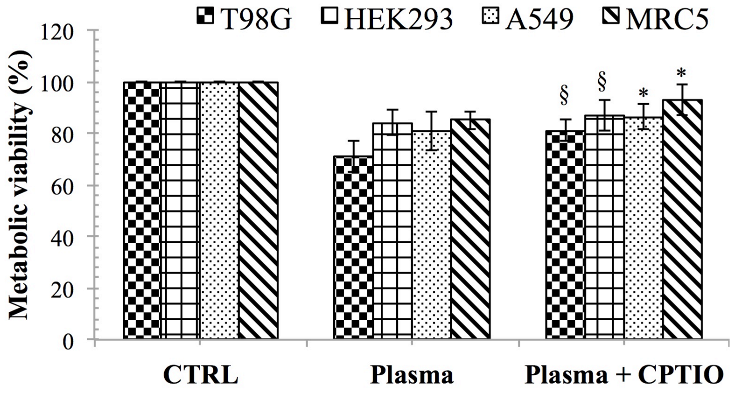

Figure S3. Response on cancer and normal cells at 24 hour after 150 s plasma exposure in the presence of 2-(4-Carboxyphenyl)-4,4,5,5-tetramethylimidazoline-1-oxyl-3-oxide (10 μM CPTIO-NO specific scavenger). Results are expressed as the percentage of living cells compared to control conditions as the mean ± SD (n=3). Student's *t*-test was performed to controls (* p<0.05, § p<0.01, **#** p<0.001).

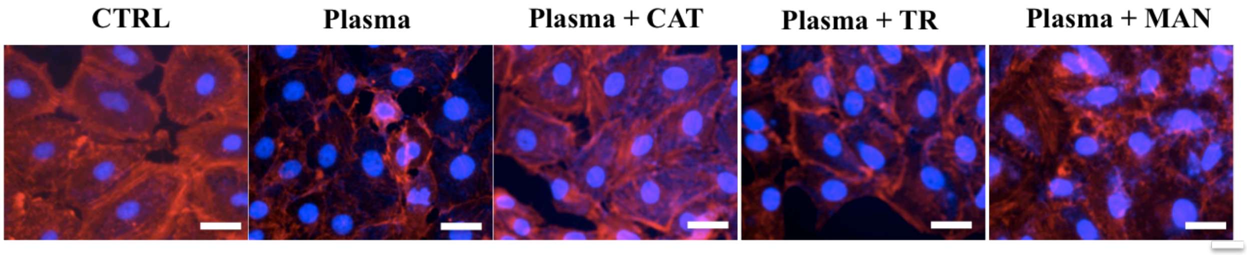

Figure S4. Immunofluorescence assays using phalloidin rhodamine were performed to visualize the cytoskeleton (F-actin); DAPI was used to label cell nucleus in A549 cells. Each figure has scale bar of 10 μm. In the plasma-treated groups, the actin filaments were distributed in the contracted cytoplasm destroying the architecture of the cytoskeleton, while scavengers restored actin structure in plasma-treated group.

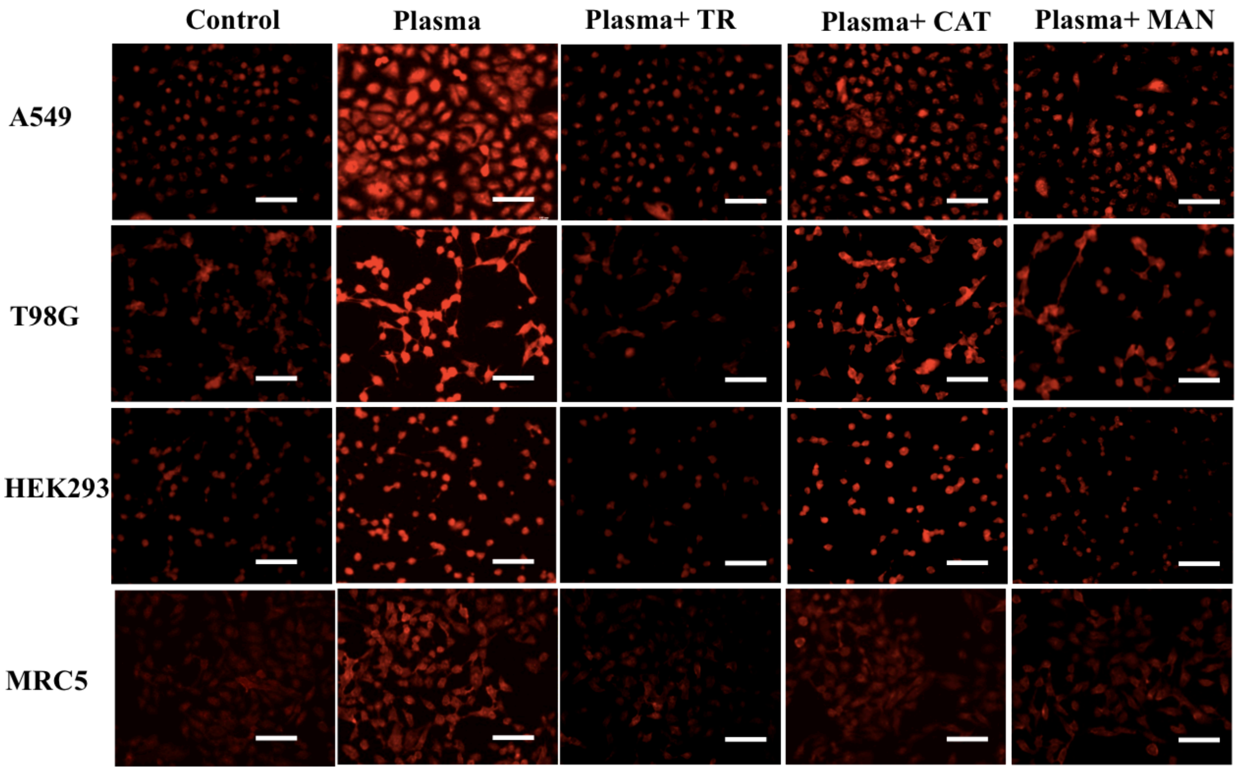

Figure S5. Dihydroethidium (DHE) labeling for measurement of superoxide anion in cancer and normal cells in plasma- and plasma plus scavengers-treated groups. Each figure has scale bar of 50 μm.

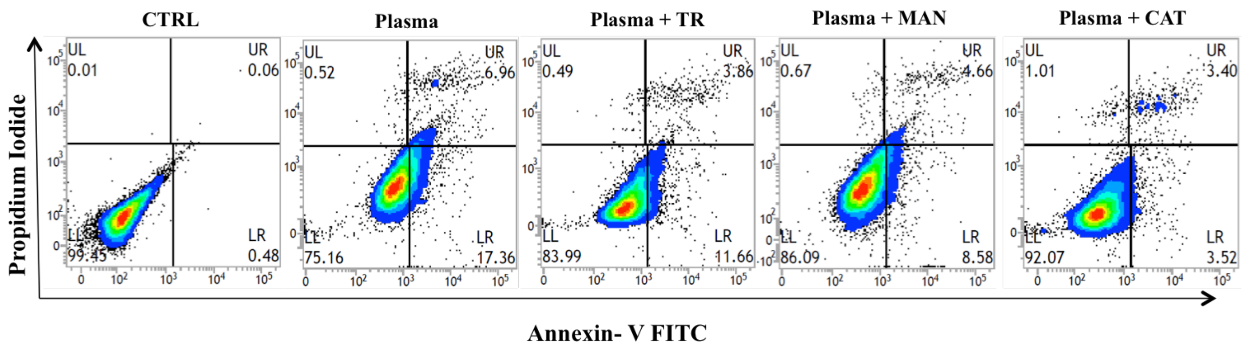

Figure S6. Apoptotic population of A549 cells treated with plasma and plasma plus scavengers groups were assessed using annexin V-FITC/PI staining and flow cytometry.

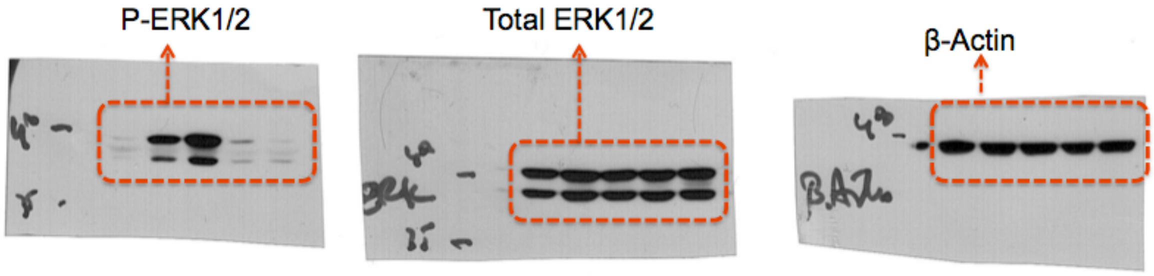

Figure S7. Full-length image of western blot result for **Figure 7**. Chemiluminescent signals were detected and recorded by exposure of the membrane to autoradiography films.

Genes	Forward primers [5–3]	Reverse primers [5–3]
β-actin	5'-CATCCGCAAAGACCTGTACG-3'	5'-CCTGCTTGCTGATCCACATC-3'
BAX	5’-CGGAATTCATGGACGGGTCCGGGGAGCAG-3’	5’-CGGAATTCGCCCATCTTCTTCCAGATGGT-3’
BAK1	5’-TTGTGAGAGCCCATTCCCAC-3’	5’-AGCCCAGAATCCCTGAGAGT -3’
Bcl2	5’-GACTTCGCCGAGATGTCCA-3’	5’-CCTCAGCCCAGACTCACATC-3’
H2AX	5’-TACCTCACCGCTGAGATCCT-3’	5’-AGCTTGTTGAGCTCCTCGTC-3’

Table S1. QPCR primer sequences for mRNA gene expression analysis.
